# Memantine-Induced Sialorrhea: A Rare Adverse Reaction and Review of Pathophysiological Mechanisms

**DOI:** 10.7759/cureus.116266

**Published:** 2026-09-15

**Authors:** Mridul Sarangal

**Affiliations:** 1 Psychiatry and Behavioral Sciences, Carilion Clinic, Roanoke, USA

**Keywords:** adverse drug events, clinical psychiatry, geriatric psychiatry, pharmacovigilance, progressive dementia, rare side effect

## Abstract

Memantine is a noncompetitive N-methyl-D-aspartate (NMDA) receptor antagonist widely utilized in the management of moderate-to-severe neurocognitive disorders, specifically Alzheimer's disease. While its common adverse effects, such as dizziness, headache, and confusion, are well documented, parasympathetic or motor-related side effects such as drooling are not listed in the standard prescribing information and remain rarely reported in clinical practice. We present a case of a 68-year-old man with a primary diagnosis of moderate-to-severe major neurocognitive disorder due to Alzheimer's disease who developed pronounced daytime drooling immediately following the initiation of oral memantine therapy. Before starting the medication, the patient had no history of excessive drooling, dysphagia, parkinsonism, or recent changes to his baseline medication regimen. Within 24 hours of taking the initial 5 mg dose, he began experiencing significant pooling of saliva, requiring frequent wiping of the mouth. A clinical evaluation revealed no facial asymmetry, resting tremor, rigidity, or signs of acute neurological deficits. Due to the strong temporal relationship between the drug administration and the onset of symptoms, memantine was promptly discontinued. The patient experienced a complete resolution of symptoms within a day of the final dose, representing a positive dechallenge. Application of the Naranjo Adverse Drug Reaction Probability Scale yielded a score of 5, indicative of a probable adverse drug reaction. This phenomenon may be driven by off-target antagonism at alpha-7 nicotinic acetylcholine receptors or the modulation of central glutamatergic signaling within the brainstem salivatory nuclei, leading to localized parasympathetic dysregulation or subclinical impairment of automatic swallowing. This case emphasizes the importance of clinical vigilance when initiating memantine. It highlights that new-onset drooling should prompt clinicians to consider a reversible, drug-induced etiology, though it should not preclude appropriate neurological evaluation to rule out acute pathology.

## Introduction

Memantine is a non-competitive N-methyl-D-aspartate (NMDA) receptor antagonist approved by the Food and Drug Administration (FDA) for the treatment of moderate-to-severe Alzheimer’s disease [1]. By partially blocking the chronic, pathological overactivation of NMDA receptors, memantine helps normalize glutamatergic neurotransmission, thereby preserving cognitive function and slowing clinical progression [2]. It is generally well-tolerated in older adult populations, with the most frequently reported adverse effects being dizziness, headache, confusion, and constipation [2].

Adverse effects involving the autonomic nervous system or isolated oral-motor dysfunction are not typically associated with memantine therapy [3]. It is important to distinguish true hypersalivation - an overproduction of saliva - from drooling, which is the impaired handling and clearance of normal salivary volumes. This distinction is particularly relevant in neurocognitive disorders, where reduced spontaneous swallowing can produce clinically apparent drooling without glandular hypersecretion. Drooling is not merely a cosmetic issue; it carries severe clinical risks, including saliva aspiration, asphyxia, and respiratory sepsis.

While excessive salivation is a well-documented side effect of other psychotropic medications, most notably clozapine, its emergence following NMDA receptor blockade is highly atypical [1,3]. Recognizing potentially rare, reversible medication-induced adverse effects is critical to prevent prescribing cascades [4]. Herein, we detail a presentation of acute drooling following the initiation of memantine in a 68-year-old man, which rapidly resolved upon discontinuation.

## Case presentation

A 68-year-old right-handed man presented, accompanied by his wife, to the outpatient psychiatry clinic for routine evaluation and management of progressive cognitive decline. Over the preceding three years, the patient experienced gradual memory loss, word-finding difficulties, and impairments in complex executive tasks such as managing finances. His functional impairment led to a primary diagnosis of moderate major neurocognitive disorder due to probable Alzheimer’s disease. He had not previously taken any acetylcholinesterase inhibitors or other cognitive-enhancing pharmacotherapy.

His past medical history was significant for essential hypertension (five years), hyperlipidemia (four years), and type 2 diabetes mellitus (three years). He had no past psychiatric hospitalizations, previous surgical procedures, history of traumatic brain injury, or history of substance abuse. His family history was non-contributory for early-onset dementia or movement disorders. Socially, he was a retired accountant living with his spouse, requiring mild assistance with instrumental activities of daily living (IADLs) but remaining independent in basic activities of daily living (ADLs).

His baseline daily medication regimen had been stable for over 12 months with no recent adjustments or discontinuations: lisinopril: 10 mg orally once daily (duration: five years), atorvastatin: 20 mg orally once daily (duration: four years), and metformin: 500 mg orally twice daily (duration: three years).

At the initial evaluation (Day 0), a baseline mental status examination revealed temporal disorientation and impaired short-term recall, yielding a Montreal Cognitive Assessment (MoCA) score of 13/30 [5]. Physical and neurological examinations demonstrated intact cranial nerves II-XII, normal bilateral facial movement, normal soft palate elevation, a preserved gag reflex, and intact baseline oropharyngeal swallowing function without baseline sialorrhea or dysphagia. Targeted motor evaluation showed no resting tremor, cogwheel rigidity, bradykinesia, gait ataxia, or other signs of parkinsonism. Vital signs were completely within normal limits: blood pressure of 126/78 mmHg, heart rate of 70 beats/min, respiratory rate of 15 breaths/minutes, body temperature of 98.2°F (36.8°C), and oxygen saturation of 99% on room air. Baseline laboratory investigations, including a comprehensive metabolic panel, complete blood count, thyroid function, and vitamin B12 levels, were unremarkable (Table 1).

**Table 1:** Baseline Laboratory Investigations

Laboratory investigation	Patient result	Reference range
Sodium	139 mEq/L	135-145 mEq/L
Potassium	4.2 mEq/L	3.5-5.0 mEq/L
Chloride	102 mEq/L	96-106 mEq/L
Bicarbonate	24 mEq/L	22-29 mEq/L
Blood urea nitrogen (BUN)	16 mg/dL	7-20 mg/dL
Serum creatinine	0.9 mg/dL	0.7-1.3 mg/dL
Fasting blood glucose	108 mg/dL	70-99 mg/dL
Aspartate aminotransferase (AST)	22 U/L	10-40 U/L
Alanine aminotransferase (ALT)	25 U/L	7-56 U/L
White blood cell (WBC) count	6.4 × 10³/µL	4.5-11.0 × 10³/µL
Hemoglobin	14.2 g/dL	13.8-17.2 g/dL
Platelets	245 × 10³/µL	150-450 × 10³/µL
Thyroid-stimulating hormone (TSH)	2.1 µIU/mL	0.4-4.0 µIU/mL
Vitamin B12	480 pg/mL	200-900 pg/mL

To target progressive cognitive decline, oral memantine therapy was initiated at a standard starting dose of 5 mg orally once daily. No previous chronic medications were discontinued. Within 18 to 24 hours of receiving the first dose (Day 1), the patient developed pronounced, continuous daytime salivary pooling and drooling. The saliva continuously spilled from the corners of his mouth, requiring the frequent use of a cloth to wipe his face. The patient remained alert and denied choking, dyspnea, nausea, odynophagia, or subjective throat tightness.

An urgent clinical assessment was conducted on Day 2 after the patient had received two total doses of memantine. Repeat examination confirmed stable vital signs (BP: 128/76 mmHg, HR: 72 beats/min) and no focal neurological deficits, dysarthria, or extrapyramidal signs. Given the clear temporal relationship, memantine was withheld due to a suspected adverse drug reaction and was not immediately substituted with an alternative cognitive agent.

Within 24 hours of drug withdrawal (Day 3), the drooling completely resolved, representing a positive dechallenge, and the patient returned to his baseline oral-motor clearance. Outpatient follow-ups conducted at two weeks (Day 14) and four weeks (Day 28) confirmed sustained resolution of symptoms, with no recurrence of salivary pooling or swallowing dysfunction while maintaining his baseline chronic medication regimen. The chronological progression is detailed in Table 2. The application of the Naranjo Adverse Drug Reaction Probability Scale yielded a total score of 5, indicating a "probable" adverse drug reaction to memantine [6].

**Table 2:** Timeline of Clinical Events MoCA: Montreal Cognitive Assessment

Time/day	Clinical event and intervention
Day 0	Routine outpatient psychiatric evaluation for cognitive decline (MoCA: 13/30). Baseline exam and labs normal. Patient initiated on memantine (5 mg) orally once daily.
Day 1	Within 18-24 hours of the first dose, the patient develops continuous daytime salivary pooling and drooling, requiring constant wiping.
Day 2	Follow-up clinical evaluation confirms normal vitals and intact cranial nerve/motor examination. Memantine is discontinued after two total doses.
Day 3	Within 24 hours of memantine withdrawal (+24 h post-drug), complete resolution of drooling was observed with a return to baseline oral clearance (positive dechallenge).
Day 14 and Day 28	Outpatient follow-up evaluations confirm no recurrence of drooling or bulbar symptoms. Baseline chronic medications continued.

## Discussion

Adverse drug reactions involving drooling are most frequently associated with medications possessing direct cholinergic agonist properties or those causing extrapyramidal symptoms [7]. The occurrence of impaired salivary clearance secondary to memantine is highly unusual. In this case, a compelling temporal correlation was observed. The Naranjo Adverse Drug Reaction Probability Scale yielded a score of 5, designating the reaction as "probable" [6].

It is highly relevant to distinguish between excess production and impaired clearance. Rather than inducing true glandular hypersecretion, NMDA antagonism might subtly impair baseline automatic swallowing the frequency or oral-motor coordination [8,9]. The precise pathophysiological mechanism remains speculative. Memantine acts as a non-competitive antagonist at the alpha-7 nicotinic acetylcholine receptor [10]. Antagonism at these autonomic ganglionic sites could dysregulate parasympathetic signaling to the salivary glands [11]. Alternatively, NMDA receptors located within the brainstem's salivatory nuclei mediate excitatory control over salivary flow [8,9]; disruption here could inadvertently disinhibit parasympathetic output.

This case has limitations. The absence of a rechallenge precludes establishing definitive causality. However, intentional rechallenge was not pursued, as it is not clinically appropriate to expose a vulnerable patient to unnecessary aspiration risks. Furthermore, while drug interactions were ruled out, the inherent polypharmacy and comorbidities common in this age group require careful consideration when evaluating any adverse event.

Crucially, acute-onset drooling may occasionally indicate significant neurological pathology, such as an acute ischemic event or bulbar palsy. This case should not imply that appropriate diagnostic evaluation can be routinely omitted when an older adult presents with sudden oropharyngeal impairment.

## Conclusions

New-onset drooling following the initiation of memantine is a rarely reported but clinically significant adverse drug reaction. Clinicians must maintain a high index of suspicion for medication-induced etiologies when patients present with acute, unexplained bulbar symptoms. Recognizing memantine as a potential cause of impaired salivary clearance can prevent prescribing cascades. However, prompt discontinuation of the suspected agent must be balanced with appropriate clinical vigilance, ensuring that severe underlying neurological evaluations are not bypassed.
